# Supplementary material for: Resolving the full spectrum of human genome variation using Linked-Reads
Source: Genome Res. 2019 Apr;29(4):635–45. doi: 10.1101/gr.234443.118 (PMC6442396; doi:10.1101/gr.234443.118)
Supplement: Supplemental Material [file supp_gr.234443.118_Supplemental_longranger-2.2.zip › longranger-2.2.tar.bz2!/lr2.2-public/lib/stan/cmdstan/stan/doc/api/html/math_2matrix_2sort_8hpp_source.html]

Stan: src/stan/math/matrix/sort.hpp Source File


|  |  |
| --- | --- |
|  | Stan  2.5.0  probability, sampling & optimization |


- Main Page
- Related Pages
- Namespaces
- Classes
- Files

- File List
- File Members

All Classes Namespaces Files Functions Variables Typedefs Enumerations Enumerator Friends Macros Pages

- src
- stan
- math
- matrix

sort.hpp

Go to the documentation of this file.

1 #ifndef STAN\_\_MATH\_\_MATRIX\_\_SORT\_HPP

2 #define STAN\_\_MATH\_\_MATRIX\_\_SORT\_HPP

3

4 #include <stan/math/matrix/Eigen.hpp>

5 #include <vector>

6 #include <algorithm> // std::sort

7 #include <functional> // std::greater

8

9 namespace stan {

10  namespace math {

11

19  template <typename T>

20  inline typename std::vector<T> sort\_asc(std::vector<T> xs) {

21  std::sort(xs.begin(), xs.end());

22  return xs;

23  }

24

32  template <typename T>

33  inline typename std::vector<T> sort\_desc(std::vector<T> xs) {

34  std::sort(xs.begin(), xs.end(), std::greater<T>());

35  return xs;

36  }

37

45  template <typename T, int R, int C>

46  inline typename Eigen::Matrix<T,R,C> sort\_asc(Eigen::Matrix<T,R,C> xs) {

47  std::sort(xs.data(), xs.data()+xs.size());

48  return xs;

49  }

50

58  template <typename T, int R, int C>

59  inline typename Eigen::Matrix<T,R,C> sort\_desc(Eigen::Matrix<T,R,C> xs) {

60  std::sort(xs.data(), xs.data()+xs.size(), std::greater<T>());

61  return xs;

62  }

63

64  }

65 }

66 #endif

stan::math::sort\_desc

std::vector< T > sort\_desc(std::vector< T > xs)

Return the specified standard vector in descending order.

**Definition:** sort.hpp:33

Eigen.hpp

stan::math::sort\_asc

std::vector< T > sort\_asc(std::vector< T > xs)

Return the specified standard vector in ascending order.

**Definition:** sort.hpp:20


---

[ Stan Home Page ]

*© 2011–2014,
Stan Development Team.*
